# Beyond the Bubble: Answer Justification, Metacognitive Monitoring, and Revision in Multiple-Choice Testing

**DOI:** 10.3390/bs16081328

**Published:** 2026-08-03

**Authors:** Malia B. Hardy, Sydney L. Kostal, Ashna Nayak, Michelle L. Rivers

**Affiliations:** Department of Psychology, Santa Clara University, Santa Clara, CA 95053, USA

**Keywords:** multiple-choice testing, assessment, metacognition, answer revision

## Abstract

Multiple-choice tests are efficient and objectively scored, but they provide limited insights into the reasoning underlying students’ responses. Requiring students to justify their answers may encourage more generative processing while also providing diagnostic information about their understanding. To examine this possibility, the present research investigated whether answer justification influences multiple-choice performance, confidence, confidence–accuracy resolution, and answer revision. In two experiments, undergraduate students studied an introductory geology text before completing multiple-choice assessments. Specifically, in Experiment 1, students completed an initial set of items, later reconsidered the same items, and then responded to new, conceptually related items. During the reconsideration phase, students either explained their reasoning before rating their confidence or simply re-answered and rated their confidence. In Experiment 2, a larger sample completed all items within a single test while changes in selected responses were unobtrusively recorded. Across both experiments, we did not find sufficient evidence demonstrating that requiring justification improved performance, confidence, confidence–accuracy resolution, or revision likelihood under the conditions examined. Nevertheless, revisions produced a positive net benefit: wrong-to-right changes occurred more frequently than right-to-wrong changes, and greater confidence was associated with a lower likelihood of revising. Moreover, exploratory analyses indicated that justification content was associated with accuracy and confidence. Conceptual-understanding justifications showed the strongest association with accuracy, and several non-guessing justification types were associated with greater confidence than guessing. Although correlational, these patterns suggest that written justifications may provide useful diagnostic information about students’ reasoning.

## 1. Introduction

A student taking a multiple-choice exam may select an answer for many reasons: genuine understanding, strategic guessing, recognition of a familiar term, or elimination of implausible alternatives. When the student is asked to justify that answer, however, the task changes. They must retrieve relevant information, evaluate alternatives, and articulate why their selected option is correct. In this way, answer justification may make multiple-choice testing more generative while also providing insights into the reasoning behind students’ responses.

The present research examined whether requiring students to justify their answers to multiple-choice questions influences test performance, confidence, confidence–accuracy resolution, and answer revision. Although prior work suggests that answer justification can improve performance and may encourage students to reconsider their answers, little is known about whether justification changes answer-revision behavior as it unfolds, whether those revisions are beneficial, or whether justification improves students’ item-level metacognitive monitoring. We also conducted exploratory analyses examining whether certain types of justifications students provided were associated with performance and confidence.

### 1.1. Multiple-Choice Testing and Answer Justification

Multiple-choice tests are widely used in classroom and large-scale educational contexts because they can efficiently assess a broad range of content and be scored objectively (National Research Council, 2001). Students may also prefer multiple-choice assessments because they perceive them as less cognitively demanding or expect to perform better on them (Dang et al., 2023). However, assessment format can shape how students prepare and respond: multiple-choice examinations have sometimes been associated with more surface-oriented approaches to studying than essay assignments (Scouller, 1998). Although well-designed multiple-choice items can assess complex thinking, selected responses alone provide limited evidence about the processes students used to arrive at an answer. Constructed-response formats can provide more direct information about students’ reasoning, and process-oriented research demonstrates that the cognitive activities reported by test-takers do not always align with those that item writers intended to elicit (Martinez, 1999; Pham et al., 2026). Thus, instructors must balance the efficiency and objectivity of multiple-choice assessment against the richer evidence of reasoning afforded by open-ended responses.

One approach to reconciling these priorities is to augment multiple-choice questions with answer justification, requiring students to explain the reasoning behind their selected responses. Early research on answer justification focused largely on student perceptions and generally found positive attitudes toward the opportunity to explain answers (Dodd & Leal, 1988; Gorrell & Cramond, 1988; Kottke, 2001; McKeachie et al., 1955; Nield & Wintre, 1986; see also Overono, 2025). Students have reported that formats allowing explanation can be less frustrating or anxiety-producing, although some have found explanations difficult or time-consuming (Nield & Wintre, 1986; Overono, 2025). Beyond these affective benefits, a central question is whether answer justification also improves learning and performance by helping reveal underlying misunderstandings, promote critical thinking, and support metacognitive awareness (Bassett, 2016; Tamir, 1990).

Answer justification may influence performance by requiring students to generate, retrieve, and evaluate information rather than simply recognize an answer. From a generative learning perspective, explaining one’s answer may promote the active construction of meaning and integration with prior knowledge (Wittrock, 1974; Fiorella & Mayer, 2016). It also resembles prompted self-explanation, which can help learners monitor understanding, identify knowledge gaps, integrate new information with prior knowledge, and focus on underlying principles (Bisra et al., 2018; Chi et al., 1989). However, whereas self-explanation typically occurs during learning, answer justification occurs at the time of testing. Justification may also support performance through elaborative retrieval and evaluation of alternatives: when students explain their selected answer, they may retrieve and connect more information than they would when simply selecting an option, making multiple-choice testing function more like retrieval practice combined with elaboration (McDaniel, 2023). Consistent with this account, generating explanations in response to questions can benefit memory, sometimes more than reading provided elaborations (Pressley et al., 1987). In addition, multiple-choice questions can induce retrieval when students consider plausible incorrect alternatives, and retrieving information about those alternatives may support later memory for related information (Little & Bjork, 2015; Little et al., 2012; Little et al., 2019). Together, these mechanisms suggest that justification may affect performance by changing how students process, monitor, and revise their answers.

Classroom-based research suggests that incorporating answer justification into multiple-choice questions can improve performance (Koretsky et al., 2016; Overono, 2025; Papadopoulos et al., 2022). For example, Papadopoulos et al. (2022) found higher performance on the subset of audience-response questions for which a short justification field was available than on questions without that field. Similarly, Koretsky et al. (2016) found that prompting students to explain their answers to thermodynamics concept questions often led them to change their responses, usually in a positive direction, although students who selected the correct answer were not always able to provide scientifically sound reasoning. Extending this work, Overono (2025) found that students in an upper-division psychology research methods course earned higher scores on multiple-choice “elaboration” questions, which required students to justify their selected answers, than on traditional multiple-choice questions across 12 course quizzes.

Although these studies provide useful classroom evidence, some used optional justification prompts, which may introduce selection effects because students decide when to justify. Requiring justification provides a more direct test of whether explaining an answer affects learning. Clark et al. (2025) provided such evidence in two controlled experiments: after studying an introductory geology chapter, undergraduates completed either a standard multiple-choice test or the same test with required brief explanations for each selected answer. Students in the answer-justification group outperformed those in the multiple-choice-only group on the immediate test in both experiments. In Experiment 2, this immediate benefit was replicated, but the delayed-test advantage after two days was numerical and not statistically significant. Overall, effects were small to medium: *d* = 0.54 in Experiment 1, *d* = 0.31 on the immediate test in Experiment 2, and *d* = 0.34 on the delayed test. However, benefits may not be universal: Wright and Wolff (2024) found that asking participants to justify why answers to multiple-choice math questions might be correct or incorrect did not significantly improve accuracy.

### 1.2. Answer Justification and Metacognition

If answer justification prompts students to evaluate the reasons for their choices, it may affect not only performance but also students’ awareness of when their answers are likely to be correct. Metacognition refers to the processes involved in evaluating and regulating one’s understanding and cognitive performance (Flavell, 1979), and in educational contexts, metacognitive monitoring is important because learners’ judgments about what they know can guide decisions about studying, reviewing, and revising answers (Dunlosky & Rawson, 2012). Monitoring is often examined by asking learners to judge their performance and comparing those judgments with objective outcomes (Nelson, 1990; Schraw, 2009). Absolute accuracy, or calibration, refers to whether confidence aligns with overall performance, whereas relative accuracy, or resolution, refers to whether learners can distinguish correct from incorrect responses. Answer justification may influence this monitoring process by changing the cues learners use to evaluate their knowledge. According to the cue-utilization framework, metacognitive judgments are based on cues available at the time of judgment rather than direct access to memory strength (Dinsmore & Parkinson, 2013; Koriat, 1997). When students explain why they selected an answer, they may attend to more diagnostic cues, such as whether they can retrieve relevant information, articulate a coherent rationale, recognize uncertainty, or rule out competing alternatives. However, cues generated during justification may vary in their diagnosticity: conceptually relevant reasoning may accompany stronger understanding, whereas vague, circular, or familiarity-based explanations may provide less reliable evidence of knowledge.

Prior research suggests that answer justification can influence confidence and metacognitive accuracy, although findings are mixed. Wright and Wolff (2024) found that justification prompts reduced confidence and overconfidence but did not make confidence ratings more diagnostic of item-level accuracy. Clark et al. (2025) found that answer justification influenced absolute accuracy, suggesting that anticipating justification may affect how students evaluate their knowledge both before and after testing. Papadopoulos et al. (2022) found that students reported higher confidence for questions that included a justification option, although the study did not examine metacognitive accuracy. These mixed findings may reflect differences in when judgments were collected and how monitoring accuracy was measured. Because confidence in the present study was measured on an ordinal 1–5 scale rather than as an estimated probability of being correct, we focused on relative accuracy, or confidence–accuracy resolution. Specifically, we examined whether students reported greater confidence for correct than for incorrect responses. This item-level approach is useful because interventions such as justification prompts may reduce overconfidence without necessarily improving the diagnosticity of confidence judgments (Wright & Wolff, 2024).

### 1.3. Answer Revision as a Metacognitive Process

Metacognitive monitoring also provides the basis for answer revision, or changes from an initially selected multiple-choice option to a later-selected option. Although only a small proportion of item responses are typically changed, many examinees revise at least one response when review is permitted. Across studies, wrong-to-right changes generally outnumber right-to-wrong changes, producing small positive-score gains on average (Coffey et al., 2024; Liu et al., 2015; Waddell & Blankenship, 1994). This pattern has been observed across multiple testing formats and academic domains (e.g., Liu et al., 2015; Ouyang et al., 2019; Waddell & Blankenship, 1994).

However, the benefits of answer revision depend on how effectively students regulate their decisions. Successful revision requires learners to monitor the quality of their initial responses and use that information to decide whether a change is warranted. Students appear to rely on cues such as perceived question difficulty, response latency, and response confidence when approaching multiple-choice questions (e.g., Higham & Gerrard, 2005; Prinsell et al., 1994; Vuk & Morse, 2012). For example, Couchman et al. (2016) found that students were more likely to revise low-confidence responses than high-confidence responses, and these revisions were generally beneficial. Similarly, Stylianou-Georgiou and Papanastasiou (2017) found that students were more likely to revise low-confidence responses and answers to difficult items, although confidence was positively associated with successful revision. Tiffin-Richards et al. (2022) further showed that confidence and perceived difficulty jointly predicted answer changing: when confidence was low, students changed answers regardless of perceived difficulty, whereas when confidence was high, they primarily changed answers to items perceived as difficult. Together, these findings suggest that answer revision is metacognitively guided, but students’ use of these cues is imperfect.

Answer revision may therefore be one behavioral pathway through which answer justification affects performance. Explaining an answer may prompt students to evaluate whether their selected response is consistent with their understanding, potentially revealing gaps, inconsistencies, or alternative interpretations that lead them to revise. Prior studies have suggested that revision may help explain the benefits of answer justification (e.g., Koretsky et al., 2016), but this possibility has not been examined directly. Clark et al. (2025) provided preliminary evidence: after testing, approximately three-quarters of participants in the answer-justification group reported that justification led them to change their responses at least some of the time. However, retrospective self-reports cannot determine how often answer changes actually occurred, whether those changes improved or impaired performance, or whether students accurately remembered their revision behavior. Thus, an important unresolved question is whether answer justification changes revision behavior as it unfolds during a test.

### 1.4. Current Investigation

The current study examined whether answer justification influences multiple-choice test performance, confidence, confidence–accuracy resolution, and answer-revision behavior. Across two experiments, participants studied an introductory geology text and then completed multiple-choice questions assessing their understanding. We asked four primary questions: Does answer justification improve performance on multiple-choice questions, including new conceptually related questions? Does answer justification affect confidence and confidence–accuracy resolution? Does it increase the likelihood of answer revision? And when revisions occur, are they more likely to improve or impair accuracy?

Experiment 1 provided an initial test of these questions using a multi-phase design in which participants first answered multiple-choice questions, later re-answered those same questions either with or without justification, and then completed new conceptually related questions. Experiment 2 used a larger sample and a single-test design in which all answer changes were unobtrusively tracked, allowing us to more directly examine revision likelihood and the consequences of revision for performance. Finally, exploratory analyses examined whether the nature of students’ justifications was associated with accuracy and confidence.

## 2. Experiment 1

Experiment 1 examined whether answer justification improved performance on previously answered multiple-choice questions and on new, conceptually related questions. Participants studied an introductory geology text, completed an initial multiple-choice test, and later re-answered the same questions either with or without answer justification before completing a related test. We predicted that the answer-justification and multiple-choice-only groups would perform similarly on the initial questions, but that the answer-justification group would outperform the multiple-choice-only group on re-answered and related questions.

### 2.1. Method

#### 2.1.1. Participants and Design

As an initial investigation of our research questions, we recruited as many participants as possible throughout the Spring 2025 academic quarter. The final sample included 105 undergraduate students from Santa Clara University who received partial course credit for participating. Participants ranged in age from 18 to 22 years (*M* = 19.20, *SE* = 0.10). The sample was 69.5% women, 28.6% men, and 1.9% gender diverse. In terms of racial and ethnic background, 41.9% of participants identified as White, 23.8% as Asian, 20.0% as Hispanic/Latino, 0.95% as Black/African American, and 13.3% as multiracial or mixed ethnicity. Participants completed the study in person, in groups ranging from 1 to 8 participants, using computers running Qualtrics software, version accessed March 2025–June 2026.

The study used a 2 × 3 mixed-factorial design, with group as a between-participants factor and question type as a within-participants factor. Participants were randomly assigned to either the answer-justification (AJ) group (*n* = 50) or the multiple-choice-only (MC-only) group (*n* = 55). Question type had three levels: initial questions, re-answered questions, and related questions.

#### 2.1.2. Materials

Participants read several passages from a geology textbook (approximately 3150 words, with accompanying diagrams; Earle, 2019). The study included 16 multiple-choice items divided into two eight-item sets (Set A and Set B). Each item in one set was designed to assess a similar underlying geological concept as a corresponding item in the other set (e.g., Set A: “What is the main difference between a mineral and a rock?”; Set B: “Which of these would belong in a mineral collection?”). We retrospectively evaluated differences in aggregate difficulty using performance on the initial test, prior to the experimental manipulation. Initial test performance did not differ significantly between Set A (*M* = 69.3%, *SE* = 2.4%) and Set B (*M* = 64.2%, *SE* = 3.1%) (*t*(103) = 1.32, *p* = 0.19, *d* = 0.26). Performance on each individual item and the complete questions are available in the Supplemental Materials.

Each set included questions assessing topics from the passages: “What is Geology,” “Minerals and Rocks,” “Fundamentals of Tectonic Plates,” and “Geological Time.” Some items were adapted from a previous answer-justification study that used the same passages (Clark et al., 2025), while others were developed for the present study.

Participants were randomly assigned to either set A or set B for the initial test and then completed the alternative set during the related-question phase.

#### 2.1.3. Procedure

**Figure 1:**
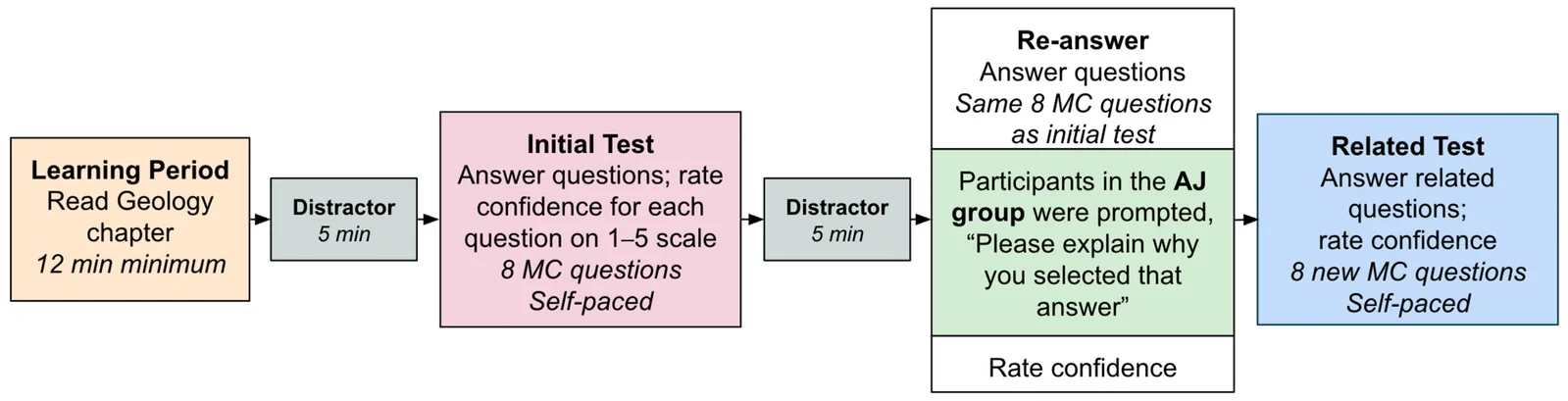
Overview of the procedure used in Experiment 1. Participants completed an initial test, re-answered the same questions either with or without answer justification, and then completed a test containing new, conceptually related questions. Experiment 2 used a different procedure in which participants completed all 16 questions within a single test while changes in their selected responses were unobtrusively recorded; this procedure is not depicted in the figure. Both experiments concluded with a global judgment of learning and demographic questions. In Experiment 2 only, participants also reported what motivated any answer revisions and completed exploratory measures assessing beliefs and experiences related to multiple-choice testing and perceptions of answer revision. AJ = answer justification; MC = multiple choice.

The procedure is depicted in Figure 1. Participants first provided informed consent and rated their prior knowledge of geology by responding to the question “How would you rate your prior knowledge of geology?” on a scale ranging from 1 (“I know nothing about geology”) to 5 (“I have extensive knowledge or formal training in geology.”) Prior knowledge was low (overall *M* = 1.93, *SE* = 0.07) and did not significantly differ between the AJ and MC-only groups (*t*(103) = 0.37, *p* = 0.71, *d* = 0.07).

Learning Period. Participants then read a chapter on geology. They were required to remain on the reading page for a minimum of 12 min but could stay longer if desired.

Phase 1: Initial Test. After the learning period, participants completed a 5 min distractor task in which they listed as many countries as they could to reduce potential recency effects. They then took an initial multiple-choice test consisting of eight questions drawn from the textbook content (e.g., “Your new roommate is a geology major. What would you be unlikely to find them studying? A. Mineral Formation; B. Weather Patterns; C. Natural Disasters; D. The Earth’s Interior”). Each participant was randomly assigned to complete either Set A or Set B questions during this phase.

After each question, participants selected an answer and rated their confidence in its correctness on a scale ranging from 1 (not at all confident) to 5 (extremely confident).

Phase 2: Re-answer. Following another 5 min country-naming distractor task, participants proceeded to the re-answer phase. They viewed the same eight questions from the initial test, along with the original answer options and their initial responses. Participants could choose to retain or change their answers.

Participants in the answer-justification group were prompted to explain their reasoning (e.g., “Please explain why you selected that answer”) before rating their confidence. Those in the MC-only group only rated their confidence after each question.

Phase 3: Related Test. In the final testing phase, participants completed the second set of eight MC questions. For example, participants who initially received Set A now completed Set B, and vice versa. After each question, participants rated their confidence using the 1–5 scale. The related questions were included to assess whether any conceptual understanding gained through answer justification transferred to new but related content within the same topic.

Final Measures and Debriefing. After completing the test, participants answered demographic questions and provided a global judgment of learning by rating how likely they were to remember what they had learned about geology that day on a 1–5 scale ranging from ‘not at all likely’ to ‘extremely likely’. This judgment did not differ between the AJ group (*M* = 2.50, *SE* = 0.13) and the MC-only group (*M* = 2.58, *SE* = 0.11) (*t*(103) = 0.48, *p* = 0.63, *d* = 0.09). Finally, participants received a debriefing that explained the purpose of the study.

### 2.2. Results and Discussion

Descriptive statistics by group and question type are presented in Table 1.

#### 2.2.1. Multiple-Choice Test Performance

Multiple-choice accuracy was analyzed at the response level using a generalized linear mixed-effects model with a binomial distribution and logit link. The model included fixed effects of group, question type, and their interaction, as well as crossed random intercepts for participants and questions. The group × question-type interaction was not significant (χ^2^(2) = 0.32, *p* = 0.85), indicating that the pattern of accuracy across question types did not differ between groups. There was also no significant main effect of question type (χ^2^(2) = 3.05, *p* = 0.22) or group (χ^2^(1) = 0.35, *p* = 0.56). Planned phase-specific contrasts indicated that the estimated odds of a correct response in the AJ group relative to the MC-only group were 1.19 for initial questions (95% CI [0.79, 1.79], *p* = 0.41), 1.06 for re-answered questions (95% CI [0.70, 1.61], *p* = 0.77), and 1.06 for related questions (95% CI [0.70, 1.60], *p* = 0.80). Thus, we did not find sufficient evidence that multiple-choice accuracy differed between groups during any phase.

#### 2.2.2. Multiple-Choice Test Confidence

Confidence judgments were analyzed at the response level using a linear mixed-effects model. The model included fixed effects of group, question type, and their interaction, as well as crossed random intercepts for participants and questions. The group × question-type interaction was not significant (F(2, 2396) = 2.72, *p* = 0.07). There was also no significant main effect of question type (F(2, 2396) = 2.76, *p* = 0.06) or group (F(1, 103) = 0.01, *p* = 0.94). Planned phase-specific contrasts indicated that the estimated AJ-minus-MC-only difference in confidence was 0.14 points for initial questions (95% CI [−0.14, 0.41], *p* = 0.33), −0.03 points for re-answered questions (95% CI [−0.31, 0.25], *p* = 0.83), and −0.08 points for related questions (95% CI [−0.35, 0.20], *p* = 0.58). Thus, we did not find sufficient evidence that confidence differed between groups during any phase.

#### 2.2.3. Timing Outcomes

We examined the time participants spent completing each question page. This measure reflected the total item-completion time, including the confidence judgment, any answer revisions, and, for participants in the AJ group during the re-answer phase, the time required to generate a written justification. Because completion times were positively skewed, response times were log-transformed and analyzed at the item level using a linear mixed-effects model. The model included fixed effects of group, question type, and their interaction, as well as crossed random intercepts for participants and questions. There was a significant group × question-type interaction (F(2, 2396) = 1242.60, *p* < 0.001), indicating that the group difference in completion time varied across question types. There were also significant main effects of question type (F(2, 2396) = 23.80, *p* < 0.001) and group (F(1, 103) = 168.00, *p* < 0.001).

Follow-up simple-effects analyses indicated that completion time did not differ significantly between the groups for initial questions (*B* = 0.09, 95% CI [−0.02, 0.20], *t*(173) = 1.65, *p* = 0.10) or related questions (*B* = −0.03, 95% CI [−0.13, 0.08], *t*(173) = −0.46, *p* = 0.65). During the re-answer phase, however, completion time was substantially longer in the AJ group than in the MC-only group (*B* = 1.91, 95% CI [1.80, 2.01], *t*(173) = −35.29, *p* < 0.001). Back-transforming this estimate indicated that AJ participants took approximately 6.72 times as long as MC-only participants (95% CI [6.04, 7.48]).

#### 2.2.4. Confidence–Accuracy Resolution

We used generalized mixed-effects logistic regression models with crossed random intercepts for participants and questions (as recommended by Murayama et al., 2014). Confidence ratings were centered within participant so that the confidence coefficient reflected whether participants were more likely to answer correctly on items for which they reported higher-than-usual confidence.

Initial confidence significantly predicted initial performance (χ^2^(1) = 47.89, *p* < 0.001). Each one-point increase in confidence relative to a participant’s own mean confidence was associated with 1.98 times greater odds of answering correctly (95% CI [1.63, 2.40]). Group did not significantly predict initial performance (χ^2^(1) = 0.54, *p* = 0.46, OR = 0.85, 95% CI [0.54, 1.32]). Most importantly, the confidence × group interaction was not significant (χ^2^(1) = 0.04, *p* = 0.85, OR = 0.97, 95% CI [0.69, 1.36]), indicating that the strength of the confidence–accuracy relationship did not differ between the AJ and MC-only groups.

We fit an analogous model predicting re-answer performance from participant-centered re-answer confidence, group, and their interaction, again including crossed random intercepts for participants and questions. Re-answer confidence significantly predicted re-answer performance (χ^2^(1) = 64.43, *p* < 0.001). Each one-point increase in confidence relative to a participant’s own mean re-answer confidence was associated with 2.24 times greater odds of answering correctly (95% CI [1.84, 2.72]). Group did not significantly predict re-answer performance (χ^2^(1) = 0.43, *p* = 0.51, OR = 0.86, 95% CI [0.56, 1.34]). The re-answer confidence × group interaction was also not significant (χ^2^(1) = 1.92, *p* = 0.17, OR = 0.78, 95% CI [0.55, 1.11]), indicating that the confidence–accuracy relationship following revision did not reliably differ between groups.

#### 2.2.5. Answer-Revision Behavior

Consistent with prior research, students revised a small percentage of their responses. Overall, 46 of 840 responses (5.5%) were revised, and 30.5% of participants revised at least one answer. Revisions were categorized as wrong to right (WR), wrong to wrong (WW), or right to wrong (RW). WR revisions occurred when participants initially selected an incorrect answer and changed to the correct answer during the re-answer phase. WW revisions occurred when participants changed from one incorrect answer to a different incorrect answer. RW revisions occurred when participants initially selected the correct answer and changed to an incorrect answer. Consistent with prior findings demonstrating that beneficial revisions tend to outnumber detrimental ones (e.g., Higham & Gerrard, 2005; Merry et al., 2021), WR revisions (2.7%) and WW revisions (1.8%) were more frequent than RW revisions (1.0%). Considering only responses that were revised, 50.0% were WR, 32.6% were WW, and 17.4% were RW.

Following McMorris and Weideman (1986), we calculated the percent gain for each participant by subtracting the number of RW revisions from the number of WR revisions, dividing by the total number of test items, and multiplying by 100. Across participants, answer changing produced a small but positive mean percent gain (*M* = 1.79%, *SE* = 0.66), indicating that revisions resulted in slightly more performance gains than losses. A one-sample *t*-test indicated that this gain was significantly greater than zero (*t*(104) = 2.69, *p* = 0.008, *d* = 0.26).

Prior research indicates that lower-confidence responses are more likely to be revised (e.g., Couchman et al., 2016; Tiffin-Richards et al., 2022). To examine this relationship, we fit a generalized mixed-effects logistic regression predicting revision behavior (0 = unchanged, 1 = revised) from participant-centered initial confidence, group, and their interaction, with crossed random intercepts for participants and questions. Initial confidence significantly predicted revision behavior (χ^2^(1) = 11.70, *p* < 0.001). Higher-than-usual confidence was associated with lower odds of revising (OR = 0.55, 95% CI [0.39, 0.77]). Thus, for each one-point increase in confidence relative to a participant’s own mean confidence, the odds of revising decreased by approximately 45%. Group did not significantly predict revision behavior (χ^2^(1) = 0.06, *p* = 0.81, OR = 1.12, 95% CI [0.46, 2.70]). The confidence × group interaction was also not significant (χ^2^(1) = 0.20, *p* = 0.66, OR = 0.86, 95% CI [0.45, 1.66]), indicating that the relation between confidence and revision behavior did not reliably differ between groups.

## 3. Experiment 2

Experiment 1 provided little evidence that answer justification improved performance, confidence, or confidence–accuracy resolution. Experiment 2 used a larger sample and an unobtrusive measure that recorded each answer selection made within a single test.

### 3.1. Method

#### 3.1.1. Participants and Design

For Experiment 2, we conducted an a priori power analysis using the small-to-medium effect observed for test performance in Experiment 2 of Clark et al. (2025), in which the AJ group outperformed the MC-only group by approximately Cohen’s *d* = 0.30. At the design stage, we estimated the required sample size using an independent-sample *t* test with a one-tailed alpha level of 0.05 and 80% power. This analysis indicated that 276 participants were needed, or approximately 138 participants per group. In the final analysis, performance was examined at the response level using a binomial logistic mixed-effects model with crossed random intercepts for participants and questions.

Participants were 298 undergraduate students from Santa Clara University (none of whom participated in Experiment 1) who were randomly assigned to either the AJ group (*n* = 148) or the MC-only group (*n* = 150). Participants ranged in age from 18 to 42 years (*M* = 19.20, *SE* = 0.10). The sample was 66.4% women, 30.5% men, and 1.7% gender diverse. In terms of racial and ethnic background, 40.3% identified as White, 22.8% as Asian, 18.1% as Hispanic/Latino, 2.0% as Black/African American, and 15.8% as multiracial or mixed ethnicity. The remaining participants did not report their racial/ethnic identity. As in Experiment 1, self-rated prior knowledge of geology was low (*M* = 1.96, *SE* = 0.04 on the 5-point scale) and did not significantly differ between the AJ and MC-only groups (*t*(296) = 0.55, *p* = 0.58, *d* = 0.06).

#### 3.1.2. Procedure

**Figure 2:**
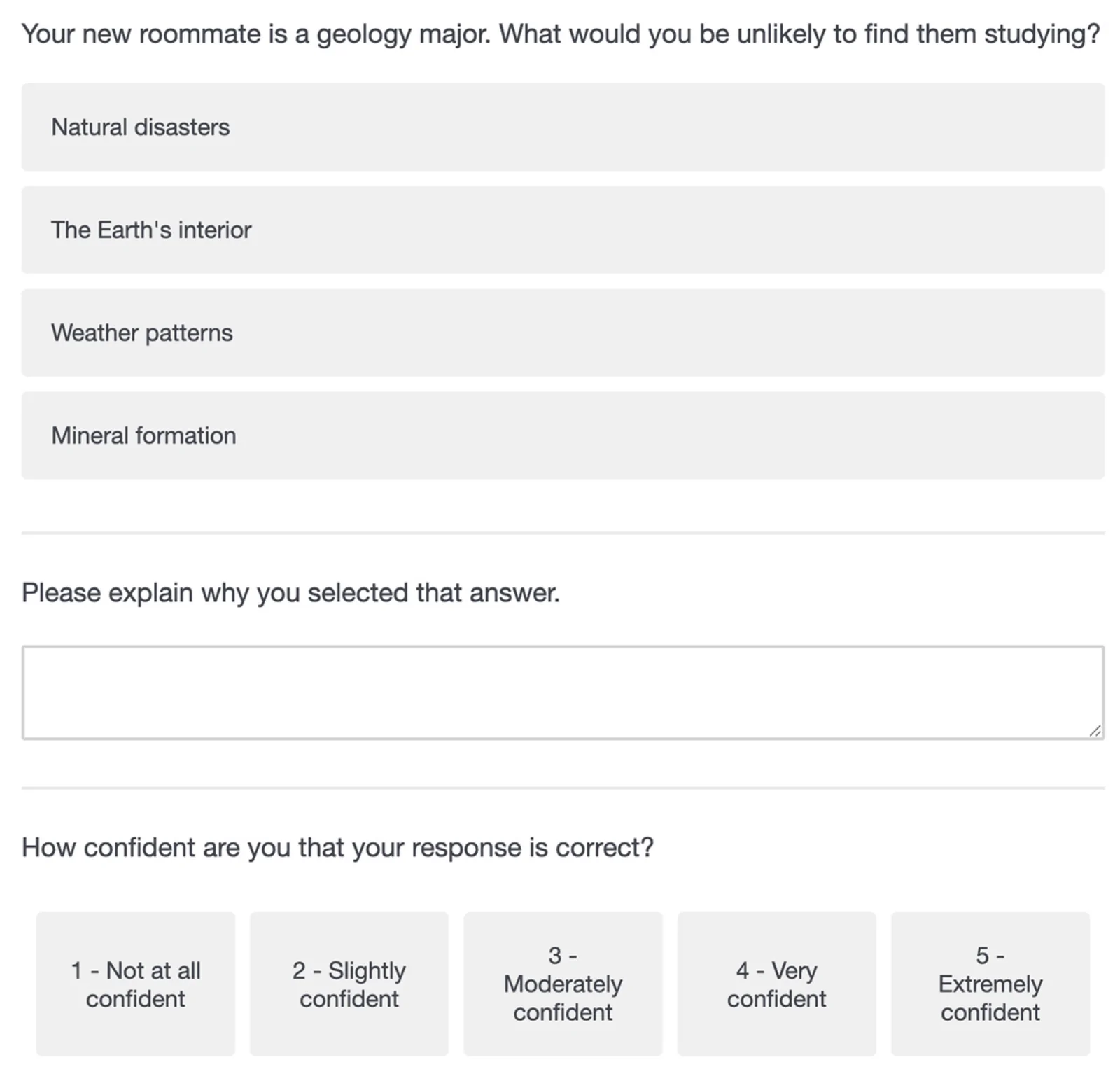
Example multiple-choice question from Experiment 2. Participants answered all 16 geology questions in a single test. In the answer-justification group, participants were prompted to explain why they selected each answer before rating their confidence on a 1–5 scale; participants in the multiple-choice-only group rated confidence without providing a justification. JavaScript recorded any answer changes participants made while responding to each question.

The materials, procedure, and data scoring were similar to Experiment 1, with the following exceptions. The initial test, re-answer phase, and related phase were collapsed into a single test. Participants saw all 16 questions (sets A and B) in a single test, rather than 8 in the initial/re-answer phase and the other 8 in the related phase. Participants in the AJ group justified each answer before rating confidence, whereas participants in the MC-only group rated confidence without providing justifications. All participants were required to rate their confidence on the same 1–5 scale. See Figure 2 for an example question. 

Unlike in Experiment 1, JavaScript recorded each answer selection in the order it occurred within a question. The first option selected was treated as the initial selection, and the last option selected before the participant advanced was treated as the final answer. Any selection of a different option after the first click was recorded as an answer-selection change. When participants made multiple changes, the complete sequence was retained. For the classification of revision outcomes, we focused on the change leading to the final answer by comparing the accuracy of the immediately preceding selection with the accuracy of the final selection. Thus, revision in Experiment 2 was operationalized as an observable change in the selected response rather than as a direct measure of deliberate metacognitive reconsideration.

Following the test, participants completed a brief exit survey before reporting their demographics. As in Experiment 1, they provided a global judgment of learning. Global judgments of learning were significantly higher for the AJ group (*M* = 2.48, *SE* = 0.07) than for the MC-only group (*M* = 2.10, *SE* = 0.07) (*t*(296) = 3.98, *p* < 0.001, *d* = 0.46). In Experiment 2 only, participants also reported what motivated any answer revisions and completed exploratory measures assessing beliefs and experiences related to multiple-choice testing and perceptions of answer revision. The exploratory measures and results are reported in the Supplemental Materials.

### 3.2. Results and Discussion

Descriptive statistics by group are presented in Table 2.

#### 3.2.1. Multiple-Choice Test Performance

Response-level accuracy was analyzed using a binomial logistic mixed-effects model with group as a fixed effect and crossed random intercepts for participants and questions. Group did not significantly predict response accuracy (χ^2^(1) = 0.64, *p* = 0.42, OR = 1.08, 95% CI [0.89, 1.32]). Thus, we did not find sufficient evidence demonstrating that requiring answer justifications affected multiple-choice test performance.

#### 3.2.2. Multiple-Choice Test Confidence

Response-level confidence judgments were analyzed using a linear mixed-effects model with group as a fixed effect and crossed random intercepts for participants and questions. Group did not significantly predict confidence (*F*(1, 296) < 0.01, *p* = 0.97, *b* = 0.003, *SE* = 0.080, 95% CI [−0.153, 0.159]). Thus, we did not find sufficient evidence demonstrating that requiring answer justifications affected participants’ confidence judgments.

#### 3.2.3. Timing Outcomes

As in Experiment 1, this measure reflected the total item-completion time rather than latency to the initial answer selection; it included answer selection, any answer changes, the confidence judgment, and, in the AJ group, written justification. Completion times were log-transformed and analyzed at the item level using a linear mixed-effects model. The model included group as a fixed effect and crossed random intercepts for participants and questions. Completion time differed significantly between groups (F(1, 296) = 969.00, *p* < 0.001). Participants in the AJ group took an estimated 2.97 times as long per question as participants in the MC-only group (95% CI [2.77, 3.19]), consistent with the additional time required to generate written justifications.

#### 3.2.4. Confidence–Accuracy Resolution

We examined confidence–accuracy resolution using a generalized mixed-effects logistic regression predicting item-level performance from participant-centered confidence, group, and their interaction, with crossed random intercepts for participants and questions. Confidence significantly predicted performance (χ^2^(1) = 212.83, *p* < 0.001). Each one-point increase in confidence relative to a participant’s own mean confidence was associated with 1.73 times greater odds of answering correctly (95% CI [1.61, 1.86]). Group did not significantly predict performance (χ^2^(1) = 0.48, *p* = 0.49, OR = 0.93, 95% CI [0.75, 1.15]). The confidence × group interaction was also not significant (χ^2^(1) = 0.01, *p* = 0.91, OR = 0.99, 95% CI [0.87, 1.13]), indicating that although confidence reliably tracked accuracy, the strength of this confidence–accuracy relationship did not differ between groups.

#### 3.2.5. Answer-Revision Behavior

Participants changed their selected response at least once on 17.5% of questions (833 out of 4768 total responses), and 89.3% of participants made at least one answer-selection change. Three hundred and twelve responses (6.5% of responses) were revised two or more times. Note that these rates are not directly comparable across experiments because Experiment 2 recorded all answer changes, whereas Experiment 1 measured changes between distinct test phases.

Focusing only on the final revision (i.e., the change leading to the final recorded answer), WR revisions were the most common (9.7%), followed by RW revisions (4.1%) and WW revisions (3.7%). Considering only responses that were revised, 55.5% were WR, 23.4% were RW, and 21.1% were WW.

As in Experiment 1, we computed percent gain. Revisions produced a positive net benefit (*M* = 5.60%, *SE* = 0.58), which was significantly greater than zero (*t*(297) = 9.61, *p* < 0.001, *d* = 0.56).

To examine whether confidence predicted answer revision, we fit a generalized mixed-effects logistic regression predicting revision behavior (0 = unchanged, 1 = revised) from participant-centered confidence, group, and their interaction, with crossed random intercepts for participants and questions. The model predicted the odds of revising relative to not revising. Confidence significantly predicted revision behavior (χ^2^(1) = 59.43, *p* < 0.001). Higher-than-usual confidence was associated with lower odds of revising (OR = 0.73, 95% CI [0.68, 0.79]). For each one-point increase in confidence relative to a participant’s own mean confidence, the odds of revising decreased by approximately 27%. Group did not significantly predict revision behavior (χ^2^(1) = 0.69, *p* = 0.41, OR = 0.91, 95% CI [0.72, 1.14]). The confidence × group interaction was also not significant (χ^2^(1) = 1.00, *p* = 0.32, OR = 1.07, 95% CI [0.93, 1.24]), indicating that the relation between confidence and revision behavior did not differ reliably between groups.

## 4. Exploratory Analysis of Students’ Answer Justifications

Despite theoretical reasons to expect answer justification to promote generative processing, we did not find sufficient evidence demonstrating that requiring students to explain their reasoning improved performance or confidence in a multiple-choice context. Contrary to our predictions and prior research (e.g., Clark et al., 2025), we did not detect higher performance among students who generated justifications in either experiment. These nonsignificant group differences motivated an exploratory examination of whether the content of students’ explanations was associated with their responses. This analysis was intended to characterize the reasoning students reported and to examine whether different justification types provided diagnostic information about accuracy and confidence. Because justification type was not experimentally manipulated, however, the analysis cannot determine whether generating a particular type of justification influenced performance. Students who understood the material better may simply have been more likely both to answer correctly and to provide a conceptually grounded explanation.

Prior research on generative learning and self-explanation provides a useful framework for distinguishing among the forms of reasoning that may appear in students’ explanations. This work differentiates explanations that reference relevant concepts, principles, and relations from responses that merely restate an answer, rely on familiarity, or offer little substantive reasoning (Fiorella & Mayer, 2016; Rittle-Johnson & Loehr, 2017). Koretsky et al. (2016) similarly showed that students’ selected answers and written reasoning did not always align. In their study, correct answers were not necessarily accompanied by scientifically appropriate explanations. Written justifications may therefore reveal aspects of students’ reasoning that cannot be inferred from multiple-choice accuracy alone. Accordingly, we coded the apparent basis of students’ explanations and examined how justification type was associated with performance and confidence.

### 4.1. Reanalysis of Clark et al. (2025)

We first examined whether justification content was associated with performance using data from the 163 participants assigned to the answer-justification groups in the two experiments reported by Clark et al. (2025). Two raters (authors S.L.K. and A.N.), masked to participants’ selected answers and multiple-choice performance, coded the written justifications using the six-category rubric shown in Table 3. The raters achieved 87.1% agreement at the response level, and discrepancies were resolved through discussion. Because some justifications reflected more than one type of reasoning, responses could receive up to two codes; 20.2% of participant–question responses received two codes. Accordingly, percentages describing the frequency of each justification type sum to more than 100%. For the analysis, each participant–question response was treated as a single observation, regardless of whether it received one or two codes, and justification types were represented using separate binary indicators. We used a generalized linear mixed-effects model with a binomial distribution and logit link to examine whether justification content was associated with item-level accuracy. Binary indicators represented whether a response contained conceptual understanding, process-of-elimination/comparison, prior knowledge, familiarity, or other reasoning. The guessing indicator was omitted so that single-coded guessing responses formed the reference profile. An additional indicator identified responses that received two codes and tested whether their outcomes differed from the combined prediction based on the two justification types they contained. Experiment was included as a fixed-effect covariate, and crossed random intercepts were included for participants and questions. The model included 1956 responses from 163 participants across 12 questions and converged successfully.

Conceptual-understanding justifications were the most common, occurring in 71.3% of responses, followed by process-of-elimination or comparison justifications in 18.1%. Guessing was coded in 9.7% of responses, familiarity in 8.9%, other reasoning in 7.0%, and prior knowledge in 5.4%. The five justification content indicators jointly improved model fit relative to a reduced model containing only experiment and double-coding status (likelihood-ratio test, χ^2^(5) = 34.84, *p* < 0.001). Relative to single-coded guessing responses, conceptual-understanding justifications were associated with higher odds of a correct response (*b* = 0.66, *SE* = 0.19, OR = 1.94, 95% CI [1.33, 2.83], *z* = 3.44, *p* < 0.001). The associations were not significant for process-of-elimination/comparison (*b* = −0.05, *SE* = 0.29, OR = 0.95, 95% CI [0.54, 1.68], *p* = 0.85), prior knowledge (*b* = −0.40, *SE* = 0.32, OR = 0.67, 95% CI [0.36, 1.25], *p* = 0.21), familiarity (*b* = 0.02, *SE* = 0.25, OR = 1.02, 95% CI [0.62, 1.68], *p* = 0.93), or other reasoning (*b* = −0.21, *SE* = 0.30, OR = 0.81, 95% CI [0.45, 1.44], *p* = 0.47).

For double-coded responses, accuracy did not differ significantly from the level predicted by the two justification types they contained (*b* = 0.14, *SE* = 0.29, OR = 1.15, 95% CI [0.65, 2.04], *z* = 0.46, *p* = 0.64). Experiment was also not significantly associated with accuracy (*b* = 0.06, *SE* = 0.17, OR = 1.06, 95% CI [0.76, 1.48], *p* = 0.73). Thus, although justification content was jointly associated with accuracy, conceptual understanding was the only individual justification type that differed significantly from guessing.

### 4.2. Analysis of the Present Study

We next pooled data from the 198 students assigned to the AJ groups across the two experiments in the present study. For Experiment 1, we focused on accuracy during the re-answer portion of the experiment, which included eight questions; Experiment 2 included 16 questions. Written justifications were coded using the same six-category rubric and procedure described in Section 4.1 and shown in Table 3. The raters achieved 81.0% agreement at the response level, and discrepancies were resolved through discussion. A total of 20.2% of participant–question responses received two codes. Conceptual understanding was the most common justification type, coded in 57.4% of responses. Familiarity was coded in 23.5% of responses, followed by elimination/comparison in 20.4%, guessing in 10.2%, other reasoning in 4.9%, and prior knowledge in 3.9%.

We again treated each participant–question response as a single observation and represented justification types using separate binary indicators. Using the same fixed-effects and crossed random-intercept structure described in Section 4.1, we fit a generalized linear mixed-effects model with a binomial distribution and logit link to examine whether justification content was associated with item-level accuracy. The model included 2768 responses from 198 participants across 16 questions and converged successfully.

The five justification content indicators jointly improved model fit relative to a reduced model containing only experiment and double-coding status (likelihood-ratio test, χ^2^(5) = 54.88, *p* < 0.001). Relative to single-coded guessing responses, conceptual-understanding responses were associated with higher odds of a correct answer (*b* = 1.03, *SE* = 0.16, OR = 2.81, 95% CI [2.07, 3.82], *z* = 6.62, *p* < 0.001). Elimination/comparison responses were also associated with higher odds of accuracy (*b* = 0.49, *SE* = 0.23, OR = 1.64, 95% CI [1.05, 2.54], *z* = 2.19, *p* = 0.02), as were familiarity responses (*b* = 0.42, *SE* = 0.17, OR = 1.52, 95% CI [1.08, 2.14], *z* = 2.40, *p* = 0.01) and responses coded as Other (*b* = 0.54, *SE* = 0.25, OR = 1.72, 95% CI [1.06, 2.78], *z* = 2.22, *p* = 0.02). The association for prior knowledge responses was not statistically significant (*b* = 0.49, *SE* = 0.28, OR = 1.63, 95% CI [0.95, 2.81], *z* = 1.76, *p* = 0.08). 

For double-coded responses, accuracy did not differ significantly from the level predicted by the two justification types they contained (*b* = −0.23, *SE* = 0.23, OR = 0.79, 95% CI [0.51, 1.24], *z* = −1.02, *p* = 0.31). Experiment was also not significantly associated with accuracy (*b* = −0.20, *SE* = 0.16, OR = 0.82, 95% CI [0.60, 1.12], *z* = −1.26, *p* = 0.21).

A parallel linear mixed-effects model examined whether justification content was associated with confidence ratings. The model used the same fixed effects and crossed random-intercept structure as the accuracy model. It included 2768 responses from 198 participants across 16 questions and converged successfully. For the likelihood-ratio comparison, the full and reduced models were refit using maximum-likelihood estimation. The five justification content indicators jointly improved model fit relative to a reduced model containing only experiment and double-coding status (likelihood-ratio test, χ^2^(5) = 455.35, *p* < 0.001).

Relative to single-coded guessing responses, conceptual-understanding responses were associated with confidence ratings that were 1.52 points higher (*SE* = 0.07, 95% CI [1.39, 1.66], *t*(2708.8) = 22.04, *p* < 0.001). Elimination/comparison responses were also associated with higher confidence (*b* = 1.07, *SE* = 0.09, 95% CI [0.88, 1.25], *t*(2663.2) = 11.28, *p* < 0.001), as were prior knowledge responses (*b* = 1.11, *SE* = 0.12, 95% CI [0.88, 1.34], *t*(2641.2) = 9.51, *p* < 0.001), familiarity responses (*b* = 1.15, *SE* = 0.07, 95% CI [1.00, 1.29], *t*(2655.5) = 15.36, *p* < 0.001), and responses coded as other (*b* = 0.98, *SE* = 0.10, 95% CI [0.78, 1.19], *t*(2650.2) = 9.38, *p* < 0.001). Thus, all five non-guessing justification types were associated with greater confidence than guessing, with conceptual-understanding justifications showing the largest adjusted difference.

In contrast, confidence ratings for double-coded responses were 1.13 points lower than predicted based on the two justification types they contained (*b* = −1.13, *SE* = 0.09, 95% CI [−1.31, −0.94], *t*(2635.1) = −11.93, *p* < 0.001). Confidence ratings were also lower in Experiment 2 than in Experiment 1 (*b* = −0.26, *SE* = 0.11, 95% CI [−0.47, −0.05], *t*(225.6) = −2.44, *p* = 0.02).

## 5. General Discussion

The present research examined whether answer justification influences multiple-choice performance, confidence, confidence–accuracy resolution, and answer revision. Across the two experiments, we did not find sufficient evidence demonstrating that requiring students to justify their answers improved test performance or metacognitive monitoring, or increased revision likelihood under the conditions examined. Answer changes produced positive net gains overall, and lower confidence was associated with a greater likelihood of answer changing. Exploratory analyses indicated that conceptual-understanding justifications were associated with greater accuracy than guessing in both data sets. In the present study, several additional non-guessing justification types were also associated with greater accuracy.

Contrary to our predictions and some prior research, we did not detect an improvement in test performance associated with answer justification, despite using similar materials to Clark et al. (2025) and increasing statistical power in Experiment 2. One possibility is that justification effects are context-dependent. Research on self-explanation suggests that explanation prompts are most effective when their content and focus align with the intended learning outcome and may be less effective under other conditions (Bisra et al., 2018; Rittle-Johnson & Loehr, 2017). Another possibility is that the confidence judgments required in both groups reduced differences between conditions. Because metacognitive judgments can be reactive (Double & Birney, 2019), confidence ratings may have prompted students in both groups to pause, evaluate their answers, and attend to cues about their knowledge, meaning the control group may not have been a purely non-reflective comparison condition. Finally, answer justification increased completion time but did not improve accuracy. Thus, in the present experiments, the additional time associated with justification did not produce a detectable accuracy benefit. Instead, what students do during that time may matter: some may use justification to retrieve relevant information and evaluate alternatives, whereas others may restate their answer, rely on familiarity, or construct a post hoc explanation. Without guidance or feedback, the additional time required for justification may not reliably support better performance.

Answer justification also did not improve confidence–accuracy resolution. Across both experiments, confidence was reliably associated with accuracy, indicating that students could somewhat distinguish correct from incorrect responses. However, this relation did not differ between groups, suggesting that explaining answers did not improve confidence–accuracy resolution. Our conclusions about metacognitive accuracy are limited to this dimension. Because confidence judgments were collected on an ordinal scale rather than as probability estimates, the present study did not assess absolute calibration or overconfidence. From a cue-utilization perspective, confidence judgments are based on cues such as fluency, familiarity, perceived difficulty, and the ability to retrieve supporting information, and the cues generated while explaining an answer may not always be diagnostic.

For example, a fluent explanation could increase confidence even when the underlying reasoning is incorrect, whereas comparing alternatives might reveal uncertainty or gaps in understanding. In the exploratory analyses, conceptual-understanding justifications were associated with both higher confidence and greater accuracy, but these associations do not show that generating such explanations improved monitoring. Students who understood the material better may simply have been more likely to answer correctly, report greater confidence, and articulate conceptually relevant reasoning. Familiarity-based justifications were also associated with higher confidence than guessing, suggesting that confidence may be elevated by cues that do not necessarily provide strong evidence of understanding. Future research could experimentally examine whether helping students evaluate the diagnosticity of different cues—for example, distinguishing familiarity from the ability to provide evidence or compare alternatives—improves monitoring accuracy.

The present experiments also contribute to research on answer revision. Across both studies, revisions produced a positive net gain, with wrong-to-right changes occurring more often than right-to-wrong changes, consistent with prior work showing that changing answers is often beneficial. However, we did not detect an increase in revision likelihood associated with answer justification, providing little evidence that justification improves performance by encouraging students to reconsider and change incorrect responses. Lower confidence was associated with a greater likelihood of answer changing in both experiments. In Experiment 1, initial confidence preceded the later opportunity to revise, consistent with the possibility that confidence guided revision decisions. In Experiment 2, however, confidence was recorded on the same page as any within-question selection changes and therefore cannot establish that confidence guided those changes.

The exploratory analyses further suggest that students’ written justifications can provide information about the reasoning accompanying their answers. Across the reanalysis of Clark et al. (2025) and the present study, correct responses were more frequently accompanied by explanations coded as reflecting conceptual understanding, and comparison-based explanations were also associated with higher accuracy in the present study. Related recent research on student-generated feedback for multiple-choice questions identified distinct forms of information—including verification, justification, explication, source use, and metacognition—and found that the complexity of this feedback was associated with academic performance (Huang et al., 2026). Although that work examined feedback accompanying student-generated questions rather than justifications for selected answers, it similarly suggests that the content of students’ written reasoning may provide information not captured by response accuracy alone.

Answer justification may be useful as a diagnostic tool because written explanations can help instructors identify differences in the apparent basis of students’ responses, including references to relevant concepts, comparison among alternatives, familiarity, or guessing. These findings suggest a potential role for written justifications in formative assessment, review activities, concept checks, and low-stakes quizzes. Given their substantial time cost, one practical possibility is to use them selectively for questions targeting conceptual understanding, common misconceptions, or applications of principles. An important direction for future research is whether modeling conceptually grounded explanations, prompting students to evaluate alternatives, or providing feedback on their reasoning can improve performance or confidence–accuracy resolution. Finally, the findings reinforce the value of teaching students about answer revision. Although students often believe that changing answers is risky (e.g., Couchman et al., 2016), the present study adds to evidence that revision is often beneficial. Educators can encourage students to revisit low-confidence responses, reread questions carefully, reconsider alternatives, and change answers when they can identify a reason for doing so.

Several limitations should be addressed in future research. Experiment 1 included a relatively modest sample, and although Experiment 2 was prospectively powered to detect a small-to-medium effect of answer justification on overall performance, it was not specifically powered for all analyses of answer revision, confidence–accuracy resolution, or interaction effects. Moreover, the relatively small number of revisions—particularly in Experiment 1—limited our ability to detect subtle group differences in revision behavior. The null findings should therefore not be interpreted as definitive evidence that answer justification has no effect across all outcomes or contexts. Replication with larger samples, more test items, and designs that provide more opportunities for revision would allow for more precise estimates of potentially small effects. In addition, answer revision is subject to selection effects because students choose which answers to revise, so the present data cannot determine what would have happened if students had been required to reconsider or revise all answers. Future work should experimentally manipulate revision opportunities to better isolate the causal effects of answer changing.

Future studies should also test whether these findings generalize beyond the present sample, materials, and testing context. Participants were recruited from a single university and studied a relatively unfamiliar introductory geology text in a low-stakes research setting. The assessments also included relatively few questions, and students’ justifications were not graded or otherwise associated with evaluative consequences. These features may limit extrapolation to authentic course examinations, more demanding or specialized courses, students with greater prior knowledge, and contexts in which the type of justification affects students’ grades or feedback. The use of unfamiliar material allowed us to examine learning under relatively controlled conditions, but the effects of answer justification may differ when students are drawing on accumulated course knowledge or responding under greater academic pressure. The answer-justification prompt used here was also relatively general, asking students to explain why they selected their answer. More targeted prompts may be needed to improve performance or metacognitive monitoring, such as prompts that ask students to explain why their answer is correct, why alternatives are incorrect, or what evidence supports their answer.

The justification-type analyses have additional limitations. Because justification type was not experimentally manipulated, these analyses cannot determine whether generating a conceptual or comparison-based explanation improves accuracy. Students who understood the material better may simply have been more likely both to answer correctly and to articulate conceptually grounded reasoning. The associations should therefore be interpreted as evidence that justification content may be a diagnostic of students’ understanding, rather than evidence that particular justification types produce better performance. These exploratory findings should be considered hypothesis-generating and require confirmation in larger, independent samples. Future studies could experimentally prompt different forms of explanation by modeling conceptual explanations, providing rubrics for justification quality, or giving feedback on students’ explanations.

## 6. Conclusions

In conclusion, answer justification may not function as a simple addition that reliably improves multiple-choice performance. Across two experiments, requiring students to explain their answers substantially increased the time spent on the task, but we did not find sufficient evidence that it improved performance, confidence–accuracy resolution, or answer revision under the present conditions. These findings suggest that producing a written explanation alone may be insufficient to produce a readily detectable benefit; its effects may depend on the reasoning demands of the questions, the content or form of the explanation generated, and the guidance or feedback students receive. 

At the same time, written justifications may have diagnostic value. Conceptual-understanding justifications were consistently associated with greater accuracy than guessing, and comparison-based justifications were also associated with greater accuracy in the present study. These findings suggest that justifications can provide information about the apparent basis of students’ selected responses. One practical possibility is to use justification selectively for questions targeting conceptual understanding, misconceptions, or comparison among alternatives rather than being required for every multiple-choice item. More broadly, considering selected answers alongside confidence, revision behavior, and written reasoning may provide a fuller picture of students’ understanding than multiple-choice accuracy alone.

## Figures and Tables

**Table 1:** Observed descriptive statistics by group in Experiment 1.

		AJ Group			MC Only	
Question Type	Initial	Re-Answer	Related	Initial	Re-Answer	Related
Performance (%)	68.2 (2.65)	69.3 (2.72)	71.0 (2.56)	65.7 (2.78)	68.2 (2.57)	69.5 (2.24)
Confidence	3.35 (0.10)	3.16 (0.11)	3.29 (0.11)	3.21 (0.10)	3.23 (0.10)	3.32 (0.10)
Completion Time (s)	18.6 (0.93)	37.3 (2.78)	16.0 (0.61)	17.0 (0.55)	5.79 (0.42)	15.9 (0.72)

Values are observed means, with standard errors in parentheses. Performance represents percentage correct, confidence was rated on a 1–5 scale, and completion time is reported in seconds. AJ = answer justification; MC only = multiple choice only.

**Table 2:** Observed descriptive statistics by group in Experiment 2.

	AJ Group	MC-Only Group
Performance (%)	66.7 (1.23)	65.2 (1.39)
Confidence	3.10 (0.06)	3.09 (0.06)
Completion Time (s)	47.8 (1.31)	16.4 (0.39)

Values are observed means, with standard errors in parentheses. Performance represents percentage correct, confidence was rated on a 1–5 scale, and completion time is reported in seconds. AJ = answer justification; MC only = multiple choice only.

**Table 3:** Justification coding categories.

Justification Type	Definition	Example
Conceptual understanding	Demonstrates understanding of the material by referencing information from the text, applying relevant concepts, or providing a conceptually appropriate example.	“I chose C because quartz is comprised of one material, which is the definition of a mineral.”
Process of elimination/comparison	Evaluates multiple response options by eliminating incorrect alternatives, comparing answer choices, or explaining why one option is better than others.	“Schist is not made from cooled lava, and I’m pretty sure granite and sandstone aren’t either, so basalt is the only one left.”
Prior knowledge	Draws on information not provided in the study materials, such as general knowledge, prior coursework, or personal experience.	“I remember learning about it in grade school.”
Familiarity	Indicates that the answer was selected because it seemed familiar, intuitive, or sounded correct, without providing a more specific rationale.	“It sounded right.”
Guessing	Indicates uncertainty and suggests that the participant selected an answer randomly or without a clear basis.	“I just picked one.”
Other	Provides no meaningful explanation, repeats the answer choice, restates the question, or gives a response that cannot be clearly classified into the other categories.	“That’s the answer.”

Responses could receive up to two codes when they reflected more than one justification type. Responses that could not be clearly classified into one or two categories were coded as Other.

## Data Availability

The original data presented in the study are openly available in the Open Science Framework at https://doi.org/10.17605/OSF.IO/H4Q2Z.

## Supplementary Materials

The following supporting information can be downloaded at: https://doi.org/10.17605/OSF.IO/H4Q2Z. References (Bandura, 1977; Foote & Belinky, 1972; Gaskins et al., 1996; Hamann et al., 2020; Harvill & Davis, 1997; Kruger et al., 2005; McMorris et al., 1987; Schwarz et al., 1991) are cited in supplementary materials.

## Abbreviations

The following abbreviations are used in this manuscript:

AJ	Answer justification
MC only	Multiple choice only
RW	Right to wrong
WR	Wrong to right
WW	Wrong to wrong
